# Pay It Forward: Analysis of a novel peer-to-peer support initiative for individuals receiving treatment for head and neck cancer

**DOI:** 10.1007/s00520-026-10776-x

**Published:** 2026-05-18

**Authors:** Matthew Mason, Justin Roe, Christine Paul, Cassandra White, Rosemary Brezmen, Claire Jeans

**Affiliations:** 1 Radiation Oncology, GenesisCare, 36 Pacific Highway, Gateshead, NSW 2290 Australia; 2 Department of Speech, Voice and Swallowing, The Royal Marsden NHS Foundation Trust, Fulham Road, London, SW3 6JJ UK; 3 Department of Otolaryngology, Head and Neck Surgery, Imperial College Healthcare NHS Trust, Fulham Palace Road, London, W6 8RF UK; 4 School of Medicine and Public Health, University of Newcastle, University Drive, Callaghan, NSW 2308 Australia; 5 Department of Medical Oncology, Maitland Hospital, 51 Metford Road, Maitland, NSW 2323 Australia; 6 Hunter Medical Research Institute, New Lambton Heights, NSW 2305 Australia

**Keywords:** Head and neck cancer, Peer-to-peer support, Distress, Radiotherapy

## Abstract

**Purpose:**

‘Pay It Forward’ is a voluntary, anonymous peer-to-peer support initiative. During chemoradiotherapy (CRT) treatment for head and neck cancer, participants receive a handwritten card of support from someone who has completed the same treatment. After recovery, individuals write their own card to assist a future person undergoing CRT. This study examined participants’ experience of the initiative and explored potential psychological benefits.

**Methods:**

This single-centre prospective study used convenience sampling and mixed methods. Structured questionnaires were completed after receiving and writing cards. Semi-structured interviews were conducted at 12 weeks following completion of CRT. Interviews were audio-recorded, transcribed verbatim, and analysed using reflexive thematic analysis.

**Results:**

Thirteen individuals were recruited (12 male, median age 66 years). Twelve reported it was positive to receive support from another patient. From thirteen interviews (mean 15 min), two main themes encapsulating the core aspects of participants’ perspectives were identified. Firstly, the therapeutic benefit and value of the card including subthemes of: instillment of hope and encouragement, personal reflection, and keeping the card safe and rereading it. The second theme described the perception that the card gave a sense of connectivity with subthemes about camaraderie, sharing with family and duty of care to future peers.

**Conclusion:**

Preliminary analysis indicates that this initiative supports patients when receiving a card during CRT, offering hope and solidarity, and when writing a card after treatment with benefits of reflection and altruism. Further research will evaluate the effectiveness and scalability of Pay It Forward across different clinical settings.

**Supplementary Information:**

The online version contains supplementary material available at https://doi.org/10.1007/s00520-026-10776-x.

## Introduction

According to The Australian Burden of Disease Study 2024, both cancer and mental health conditions were among the leading causes of disease burden in Australia [1]. The lived experience of individuals diagnosed with cancer is frequently characterised by insufficient psychological support, with systemic shortcomings failing to address emotional and existential needs across the continuum of care [2]. The human crisis in cancer: a Lancet Oncology Commission highlights that despite the significant scientific and technical progress in oncology, people affected by cancer often feel unheard and under supported [3].

In Australia in 2021, there were 5097 new cases of head and neck cancer (including lip) diagnosed with this predicted to rise in 2025 to 5577 new cases [4]. For many patients with locally advanced mucosal head and neck cancer (HNC), concurrent cisplatin-based CRT is considered a standard of care [5]. It is recognised that the physical and functional toxicities of CRT, such as radiation dermatitis, mucositis, dysgeusia, dysphagia and pain, can be overwhelming during and after treatment [6, 7]. The psychological impacts of CRT may also be challenging, with several studies suggesting that the rates of psychological distress and depression increase as patients progress through treatment [8, 9]. Nielson et al. (2013) reported an increase from 15 to 29% of probable depression rates between pre-treatment and 3 weeks post treatment, associated with the morbidity of both tumour and treatment-related factors [9].

Psychological support can be provided by a range of individuals including doctors, nursing staff and allied health professionals. Professionally led supportive interventions such as cognitive behavioural therapy and psychoeducation have been studied in HNC, although the evidence base is weak [10, 11] and the challenges of making psychological support accessible and available are recognised [12].

The potentially less-costly approach of peer-to-peer support, complementing traditional health care services, has been demonstrated to improve quality of life improving empowerment and coping [13]. However, there is limited evidence regarding peer-to-peer support and quality of life for people with HNC [14].

An inexpensive and low burden version of peer-to-peer support is the ‘Pay It Forward’ initiative developed and implemented by the principal investigator who felt that there was a gap in the emotional and mental support available to patients undergoing head and neck cancer treatment. This is a voluntary and anonymous process where a person with HNC receives a handwritten card of support during their CRT from someone who has previously completed the same treatment. After completing and recovering from their own treatment, they write their own card which is passed onto a future individual undergoing CRT. To our knowledge this intervention is the first of its kind both nationally and internationally.

### Project aim

The aim of this pilot study was to gain insights into the patients’ experience and perceptions of the Pay It Forward initiative and explore the potential psychological benefits.

## Methods

### Study design

This single-site, prospective study used a mixed-methods design. Quantitative data collected described participant demographics and tumour characteristics. Structured electronic questionnaires, using REDCap, were completed 2 weeks after receiving and writing a Pay It Forward Card. These time points were selected following pilot testing where observations and anecdotal reports from patients suggested the intervention may confer psychological benefits when both receiving the card and writing a card. Semi-structured interviews were performed 12 weeks following completion of CRT and subsequently analysed using reflexive thematic analysis.

### Ethics

Ethics approval was obtained from St Vincent’s Hospital Human Research Ethics Committee (2023/ETH01372). The procedures used in this study adhere to the tenets of the Declaration of Helsinki.

### Participants and setting

The study was conducted at GenesisCare, a radiation oncology centre based in Newcastle, NSW. All patients with newly diagnosed head and neck malignancies were screened for eligibility at initial consultation. Inclusion criteria were (1) age > 18 years, (2) histological diagnosis of mucosal head and neck malignancy, (3) concurrent CRT (70 Gy in 35 fractions) with curative intent offered, and (4) capacity to consent. Consecutively presenting eligible patients were invited to participate, forming a convenience sample.

A clinical research coordinator provided written information at the radiotherapy CT simulation appointment. Informed consent to participate, including consent for publication, was obtained at a later appointment.

### Intervention

**Fig. 1:**
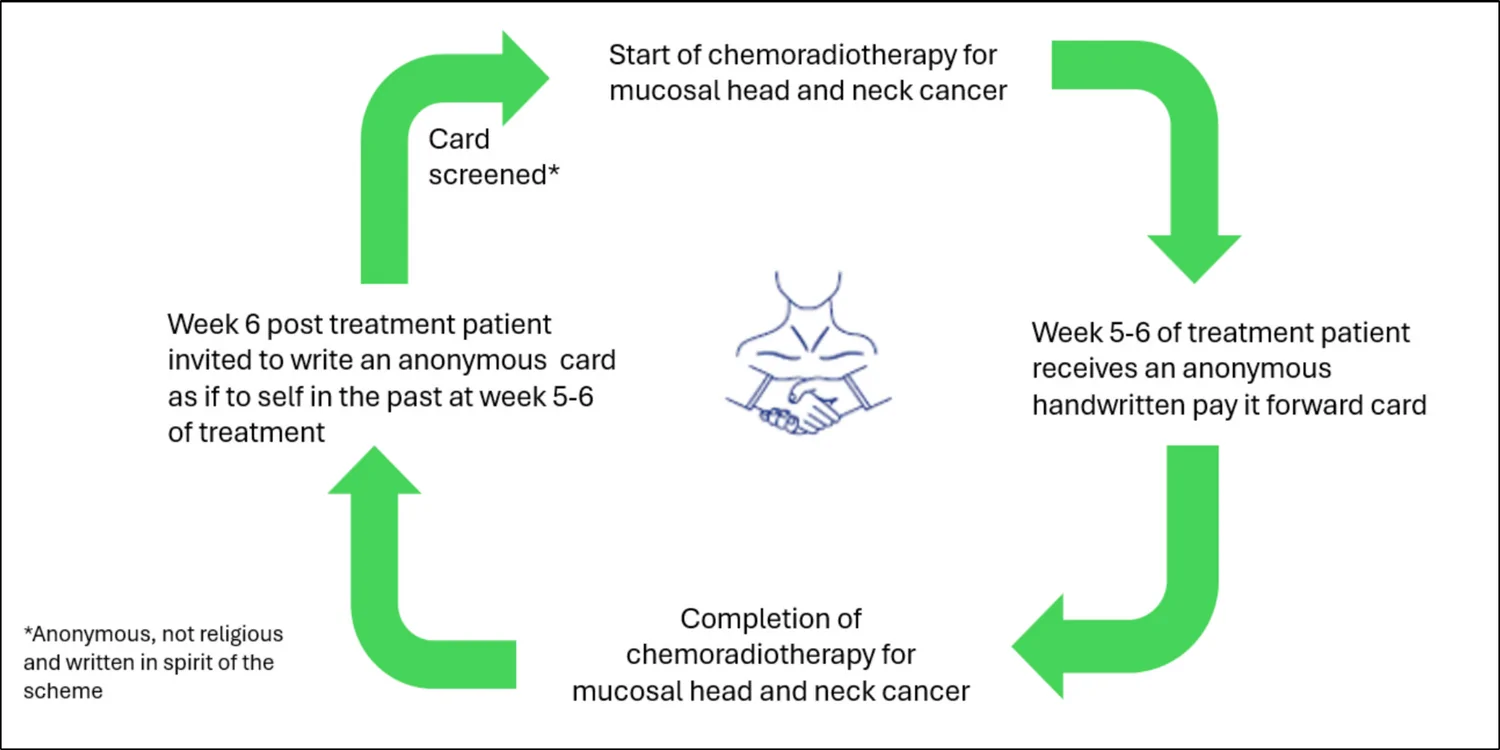
Pay it forward initiative intervention

Pay It Forward is an investigator-led anonymous peer-to peer support initiative. During CRT for mucosal HNC, individuals receive a handwritten card of support, typically at week 5–6, during their 7-week course of CRT from someone who has previously completed the same treatment. Following completion of their own treatment, typically at 6 weeks post treatment, a blank card with a short instruction message is provided (Supplementary file 1). They are invited to write their own card which is then passed to a future patient. This is an anonymous card, written as if to oneself in the past. The cards typically include an acknowledgement of the shock of diagnosis and the difficulty of treatment; there are words of encouragement noting progress is achieved each day and a sense of purpose beyond treatment. Each card is unique, but all have conveyed a sense of care, warmth, and solidarity. Cards are screened to ensure anonymity, that they avoid religious content and to confirm they are written in the spirit of the initiative before being passed on to the next patient (Fig. 1).

### Procedures

Implementation of Pay It Forward commenced at the primary site in 2022. Participants were prospectively recruited between December 2023 and March 2025. Participant demographic characteristics and disease characteristics were recorded. At week 5 of CRT, participants received a handwritten Pay It Forward card. Two weeks after receiving the card, a structured questionnaire with closed-ended questions using categorical ordinal responses was completed. This procedure was repeated 2 weeks after writing a Pay It Forward card (6 weeks after completion of treatment).

At 12 weeks following completion of CRT, one-on-one in-depth semi-structured interviews were conducted by a clinical psychologist (LR). Open-ended questions were used, and participants were provided with an interview guide in advance (See supplementary file 2). Additional time was allocated post interview for the participants to debrief and/or for the provision of psychological support if needed.

Standards for reporting qualitative research were considered when drafting this manuscript [15].

### Author/interviewer positionality

The first author and principal investigator (MM) is a Radiation Oncologist who practices as a head and neck specialist at the primary site. He conceived, developed, and implemented the Pay It Forward initiative. He is known to 12 of the 13 participants and is the physician responsible for their care. The other three authors involved in the analysis comprise a clinical nurse specialist (RB, who assists with implementation of the initiative and care at the primary site and is known to all of the participants), a speech pathologist (CJ, previously practicing at the primary site and known to four of the participants) and a speech pathologist (JR, who had no interactions with and is not known to any of the participants). The interviews were conducted by a clinical psychologist (LR) who practices at the primary site and was independent of the initiative, its design and implementation, and the research team.

### Data analysis

High-quality audio recordings were transcribed verbatim by MM. These audio transcripts were checked for accuracy by (CJ, RB, and JR). Reflexive thematic analysis was undertaken using the principles and steps recommended by Braun and Clarke [16].

Four authors (MM, CJ, RB, and JR) engaged in a collaborative, iterative approach to thematic analysis. Meeting online, on three occasions, de-identified transcripts were simultaneously and independently reviewed noting patterns, keywords and phrases and potential categories. These observations were discussed collectively to identify emerging patterns and candidate themes. Following these meetings, two authors (MM and CJ) undertook detailed coding, reviewing and refining the themes developed during the initial group discussions. After revision and refinement, feedback was sought from all authors to finalise the thematic framework and ensure coherence and credibility. Finally, the refined themes were presented to participants for member checking.

### Participant checking

Participant checking was conducted to verify the thematic analysis aligned with the participants’ perspectives. A summary of the interpreted themes was shared with the participants, with a questionnaire inviting feedback as to the accuracy of the interpretation. Participants were asked whether anything should be added to better reflect their personal experiences of the Pay It Forward initiative (Supplementary file 3).

## Results

Thirteen of fourteen eligible participants invited to join the study consented to participation. Table 1 details the participant demographics and tumour characteristics. Twelve of the participants were males with a median age of 66 years. The primary site was predominantly oropharyngeal squamous cell carcinoma, and twelve of thirteen cases were p16 positive.

**Table 1:** Participant characteristics *n* = 13

Characteristics	
Age, median (range) years	66 (42–77)
Gender	
Male	12
Female	1
Lives alone	
Yes	1
No	12
Distance from home, median (range) Km	15 (5–414)
History of anxiety depression reported	
Yes	2
No	11
Primary site	
Oropharynx	9
Unknown primary	4
T stage	
0	4
1	2
2	3
3	3
4	1
N stage	
1	10
2	3

### Questionnaire data

Structured questionnaire responses with respect to both the receiving and writing of cards are presented in Table 2. Many participants related receiving the card gave them hope that things would get better which for most was not just momentary. A moderate or major duration of effect lasting several hours or days being reported in 61.5% of respondents. Twelve participants reported it was positive to receive support from another patient with 8 strongly agreeing and 4 agreeing this felt good.

**Table 2:** Structured questionnaire responses

	Strongly agree/agree	Neither agree nor disagree	Disagree/strongly disagree
**Receiving (*n* = 13)**			
Receiving this card gave me hope that things will get better	10	2	1
Receiving this card had an influence on me	10	2	1
It felt good to receive support from another patient	12	0	1
**Writing (*n* = 12)**			
I found it easy to write this card and share my experience	11	0	1
Writing this card allowed me to reflect on my experience and assisted my recovery	10	2	0
It felt good to support another patient	12	0	0
**Duration of effect following receipt of the card**			**(*n* = 13)**
Major-term effect (lasting for several days)			3
Moderate-term effect (lasting several hours)			5
Short-term effect (lasting a few minutes)			2
The card did not influence me			3

P2 “Receiving the card gives a timely reminder that the treatment process has nearly ended. It helps strengthen the determination to make it through, knowing that others have gone before…”.

Among writers, with 12 of 13 respondents, many reported finding the process easy and felt that it assisted their recovery through reflection. All respondents agreed it felt good to support another patient.

### Interview data

Thirteen semi-structured interviews were conducted with participants to explore their experience participating in the Pay It Forward initiative. Interviews ranged from 4 to 23 minutes in duration (mean 15 min). There were no repeat interviews. Thematic saturation and sufficient information power were considered reached after ten interviews, but all thirteen interviews were analysed for completeness and out of respect for the participants who had completed interviews.

**Fig. 2:**
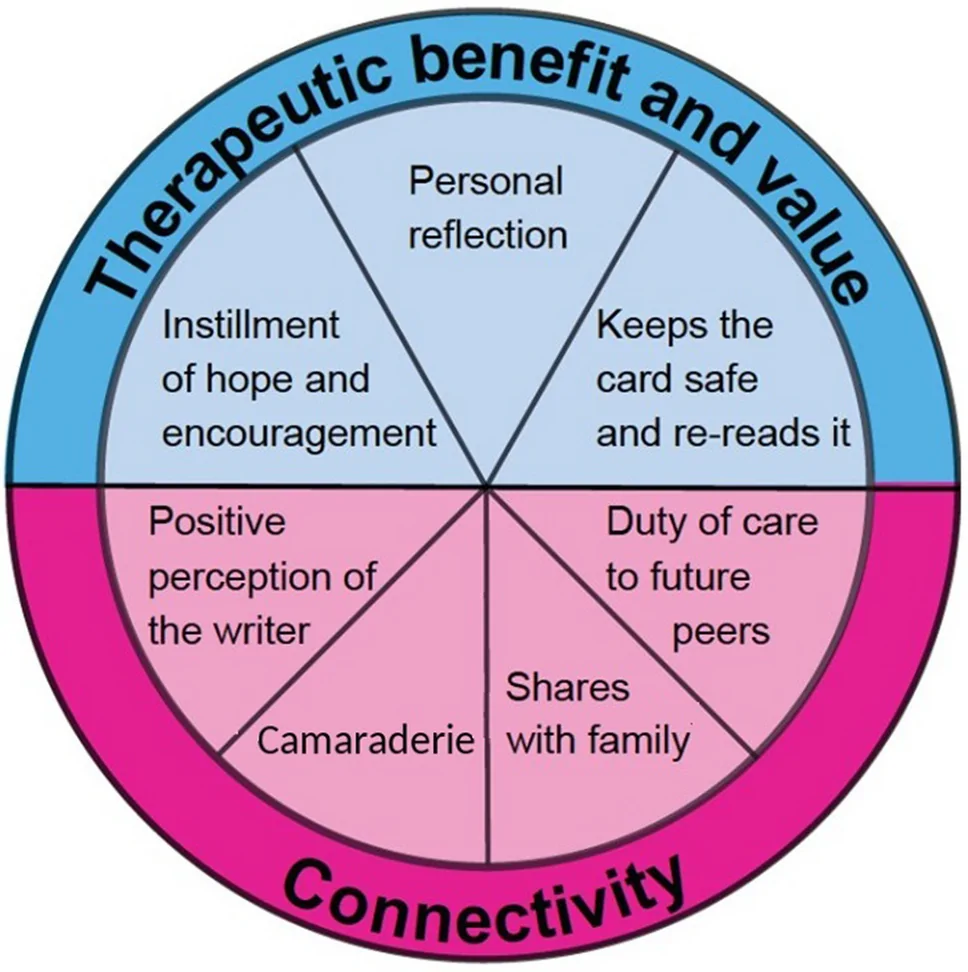
Themes and subthemes

Two main themes that encapsulated the core aspects of participants’ perspectives with subthemes were identified (see Fig. 2). To ensure anonymity, non-identifying numbers are used to cite quotes. Twelve participants responded confirming the interpretation (with themes and subthemes) very accurately (*n* = 11) and somewhat accurately (*n* = 1) reflected their experience.

## Theme 1. Therapeutic benefit and value

Therapeutic benefit and value of the Pay It Forward process were described as instillment of hope and encouragement, and personal reflection. The handwritten card was described as if a tangible symbol of thought, care and a physical token of empathy. The act of receiving and producing the card may also be significant for some; a gesture that connects peers through a shared experience. Some participants valued the dynamic process of the initiative and considered the message they received at different time points/in different contexts along their treatment journey sometimes associating different meaning and significance. Writing the card for some created positive feelings associated with altruism.

### Subtheme 1.1. Instilment of hope and encouragement (reading)

At a time of both physical and psychological vulnerability, some participants expressed that receiving the card gave them hope for the future; that there would be an improvement in their well-being. Words of encouragement and context of the challenges to be faced before the start of the recovery process were referenced. Participants felt less alone, reassured and empowered to complete their treatment. Some participants noted they were disappointed the card was not what they had expected.

P1: “It made me feel that other people had gone through it and got better… so it just made me believe that there was light at the end of the tunnel.”

P7: “To hear their experiences and um they’re sort of sharing words or encouragement. That felt very…yeah it made me really happy um and very reassuring I suppose.”

P10: “Going through all the symptoms and it goes on day after day……and it really does affect you. You know you’ve just got to….and the card really helped me feel a bit more positive about the whole thing…. Looking forward to the future.”

### Subtheme 1.2. Personal reflection (writing and reading)

Participants reflected on their treatment and recovery and in turn, it made them realise how they had improved and the challenges that they had overcome. Some considered practical aspects of their journey that could be forwarded on to help others. When engaged in writing the card about anticipated improvements one participant expressed that the process affirmed the reality that improvement and recovery were already happening. One participant referenced that reading the card made him reflect on his current situation and consider how the treatment process affected others.

P8: “It makes you think about where you’ve been and where you’re at and where you’re going.”

P9: “It was more the mental side when I was up to writing the card rather than the physical discomforts and er tiredness and all that sort of thing. So, it was good to yeah reminisce think back, think back to the journey I’d been on and what I’d overcome.”

P10: “Putting it down in words and expressing myself about how, what I’ve gone through erm and at that stage I did write it things started to improve. So, it’s not as if I’m going ‘don’t worry about it, things will get better’, and it wasn’t getting better…for me it was actually getting better.”

P3: “It was just about the process I was going through, but the card indicated that it was more, like how other people had been affected, especially family and close friends, so that put a bit more of a perspective on it.”

### Subtheme 1.3. Keeps the card safe and rereads it (reading)

Whilst not all the participants kept or valued the card, most participants found a safe, familiar and accessible location for the card during and for several months after treatment completion. One participant discarded the card within 2–3 weeks of receiving it. Another participant suggested returning to the card at different time points may provide benefit as their perception changed. Many revisited the card they had received at the time of writing their own card.

P4: “I’ve still got it. Erm Yeah, I’ve got…I’ll show you there’s one I got and one I wrote, and I’ll probably keep them forever I’d say.”

P6 “To me the card was not of great value in that it was a bit er like a lot of erm stuff you read on the internet, just trying to… saying things that are fairly obvious I would say…… I kept it for about three weeks then I threw it away.”

P9: “So that’s probably something erm that could be mentioned when they do get the card, so just don’t tuck it away, just have a read of it sort of once a week or something and maybe it may trigger something or help something in their recovery.”

## Theme 2. Connectivity

Participants described the card in terms of elements of connectivity to their peers. They reported support from someone with success in a shared experience makes the recovery more tangible (as compared to support from family, friends, etc.). Sharing the card with family, and validation of their experience to themselves and their loved ones was also expressed. The importance of being a person rather than just a patient/disease was conveyed. It made some feel seen and heard—‘I was flattered that maybe my thoughts may have mattered about another person.’

### Subtheme 2.1. Camaraderie/Peer-to-peer solidarity (reading)

Participants referenced how the card made them feel that they were not alone and reduced their sense of isolation. There were similarities in how they perceived the author of the card they received with themselves. Some felt empathy from their predecessor and some expressed empathy for future peers. This theme suggests that this insurmountable journey becomes more achievable knowing another person has gone through this ahead of them. This was considered peer-to-peer support on their own terms, when they want it.

P2 “Whoever wrote this, it could have well been me writing it…I could identify with them that’s for sure, because as I said, if I changed the age, I could have written it myself, so we have a lot in common.”

P5: “I think it’s nice to know that by receiving the card or as doing the card you’re telling someone else hey I’m out here and I’m in the same boat they’re in. They’re receiving it saying hey I’m getting this from someone in the same boat as me. I’m not alone.”

P10: “I was relieved to get it… so I could be able to read how someone else has handled it… because it’s a real personal thing. You know my wife was there all of the time, but they don’t know. You know you can explain to them but yeah er they because it’s a personal thing erm no one really knows unless they’re actually going through it.”

### Subtheme 2.2. Positive perception of the writer (reading)

Participants appeared to have a positive impression of the writer of the card that they had received even in the absence of any direct contact. They held them in high regard and wanted to emulate their predecessor when writing themselves. Patients are physically and emotionally challenged by the treatment, and it was recognised that revealing emotions/vulnerability can be difficult. Many felt the person writing their card was strong, nice and caring.

P1: “I felt they were strong… they came across as if they were a strong person.”

P8: “A fair bit of gratitude too. Yeah. Felt very grateful for the other person putting in the time and erm, you know, sharing their words of encouragement. So, it was very nice.”

P12: “You’re grateful that someone’s taken the time to do it. You know and the fact that there doing it because they want to help you, you know, that’s, that’s clearly evident.”

### Subtheme 2.3. Duty of care to future peers (writing)

Participants expressed the need to do no harm to future patients. Many felt daunted/pressured to write a card, wanting to replicate the positive influence that had been impactful for them personally—‘make them feel positive like I felt when I received mine.’ Participants reported that they took time and care to create their card. Some provided practical advice. Often, the card they had received was used as a starting point/template.

P5 “I felt very privileged to be even taking part in this to be honest with you…it’s a chance where I’ve experienced something that someone else is going to experience and just not only give them a little bit of encouragement but also what I what I was trying to do was…this is going to happen but don’t worry, it'll be ok you know.”

P6: “You don’t want to write something that’s going to make them depressed and tell them how bad it is.”

P12: “I really feel for somebody you know who is going to go through what’s coming because, because it’s not going to be pretty… the last thing you want to do is like I said scare the daylights out of them. But erm yeah, I think anything that you could do to help somebody go through it er well that’s got to be a plus.”

### Subtheme 2.4. Sharing with family (reading and writing)

Some participants showed the card to their family. To some, the card felt like a very personal gift. Showing the card to family validated their experience to themselves and their family. When writing, some participants drafted and reviewed their card with their family. The card may act as a conduit to encourage patients and family members to discuss the diagnosis and treatment journey/challenges.

### Practical aspects of the initiative (*n* = 12)

All twelve participants preferred a handwritten format for Pay It Forward with most preferring the current card (*n* = 10). A minority wanted a slightly larger card (*n* = 2) or preferred a letter (*n* = 2). Most respondents (*n* = 7) preferred the current time to receive the card (week 5–6) with one participant suggesting they would prefer the card at the start of treatment (week 1–2), 2 participants preferring mid treatment (week 3–4) and 2 participants considering the end of treatment (week 7) would be a good time to receive the card. Most participants (*n* = 9) felt the current time to write the card was appropriate. Two participants would want to write the card straight after treatment and one participant later at 3–6 months. Six preferred anonymity and six were happy to be identified.

## Discussion

Pay It Forward is a novel initiative that potentially provides benefit to patients receiving CRT for HNC in several forms and at several stages of the treatment and recovery journey. There is the process of receiving/reading a card from a peer who has previous experience of the same treatment and its challenges, and the process of reflection/writing a card for someone in the future. These two processes combined may have synergistic benefits which will be explored further in the future. The card can be considered as a personal and physical gift or token. It may have a momentary positive effect, or for some be a dynamic process influencing them at different time points as they revisit the words of encouragement from a peer or reflect on their experience.

The advantages of this scheme are that it is simple, easily reproducible, and it does not require significant resources or financial input. This initiative could also be easily integrated into the care of patients in other oncological settings.

Brief contact interventions (including crisis cards, postcards, and letters) have previously been identified as potentially relevant with respect to self-harm or suicide prevention programmes; however, they have not been recommended for widespread clinical implementation [17]. Like these initiatives, Pay It Forward consists of cards carrying messages of support. Pay It Forward however differs in its target population, source, message format, and content as well as likely mechanism of effect. In the context of suicide prevention, the postcards came from a clinician/institution, whereas Pay It Forward comes from a peer with a lived experience of the same challenge. Rather than unidirectional and received post crisis, Pay It Forward is a regenerative reciprocal initiative with the message of support received during treatment at the time of distress. Whilst the former initiative conveyed someone cares, Pay It Forward conveys someone understands. The mechanism differs with postcards considered in part to act by increasing mental health service engagement and maintaining connection [18], whereas Pay It Forward may work through restoring hope, providing encouragement and reducing isolation when cards are received. In addition, there is the benefit of writing the card which may have independent or synergistic benefit.

The majority of participants in this study were male, which aligns with the broader local HNC patient cohort. Men are generally less likely to seek social or peer support and tend to engage less in supportive care initiatives [19]. Pay It Forwards’ non-contact form of support may be particularly well suited to male patients. Conformity to masculine gender norms, including the need to manage emotions privately and avoid displays of vulnerability, has previously been demonstrated to be a significant barrier to men seeking help for cancer-related symptoms [20]. Unlike traditional support groups, Pay It Forward requires no face-to-face displays of emotions, no group participation and no direct display that the individual is experiencing or has experienced distress.

We also propose that surprise or unexpected kindness may be an active component of Pay It Forward. In clinical practice, the handwritten cards are received without forewarning, and this unexpectedness may amplify the emotional impact. Unexpected events have been demonstrated to carry greater emotional impact compared to anticipated ones [21]. In the setting of this trial, the card was expected and for some this appears to diminish or possibly changed the effect. The meaningful benefit demonstrated in this trial therefore could be interpreted as strengthening the case for clinical implementation where the element of surprise is preserved. Future investigations could compare cohorts of patients who are informed in advance of the exact support intervention with those who are not.

While many participants in the questionnaire reported they felt it was easy to write a card and share their experience, interviews revealed several felt daunted and pressured to ensure that they did a good job.

Limitations include that this study was conducted at a single small private centre and the lack of a control comparison, may limit the generalizability of the findings and the scientific rigour. The average interview duration of 15 minutes (range 4–23 min) is acknowledged as relatively brief for in depth semi structured interviews and may have limited the depth of exploration or participants’ experiences. Although it was considered that information saturation was reached after 10 interviews, the notably shortest interview, 4 minutes, warrants reflection as to whether this could yield qualitative comparable data to longer interviews. It is anticipated that insights from this preliminary work will inform and strengthen future work providing a foundation for deeper participant experience exploration.

Future research across different settings is needed to determine if this initiative may be effective, applicable at other centres and for other treatment cohorts. The perception of caregivers and staff will also be explored. It is recognised that bias is a limitation with steps taken to minimise this. An independent researcher who was not directly involved with patient care participated in the verification of transcriptions and analysis. Themes were developed through consensus in accordance with established qualitative research guidelines and interviews were conducted by a psychologist who was not an investigator, study designer or involved in the Pay It Forward initiative.

## Conclusion

This study suggests that Pay It Forward, a novel peer-to-peer support initiative, incorporates a personal, human touch that helps individuals with HNC during their treatment and recovery journey. While these findings are promising, further research is required to evaluate the effectiveness and scalability of Pay It Forward across different clinical settings.

## Supplementary Information

Below is the link to the electronic supplementary material.

ESM 1 (DOCX 1.40 MB)

## Data Availability

The qualitative data generated and analysed during this study consist of confidential interview transcripts that contain potentially identifying or sensitive participant information. Due to the nature of these data the full transcripts cannot be publicly shared. De-identified excerpts relevant to the study’s findings are included within the article. Additional de-identified materials may be made available upon reasonable request to the corresponding author.
