# Supplementary material for: Pay It Forward: Analysis of a novel peer-to-peer support initiative for individuals receiving treatment for head and neck cancer
Source: Support Care Cancer. 2026 May 18;34(6):556. doi: 10.1007/s00520-026-10776-x (PMC13183689; doi:10.1007/s00520-026-10776-x)
Supplement: Supplementary file 1 — (DOCX 1.40 MB) [file 520_2026_10776_MOESM1_ESM.docx]

Supplementary file 1


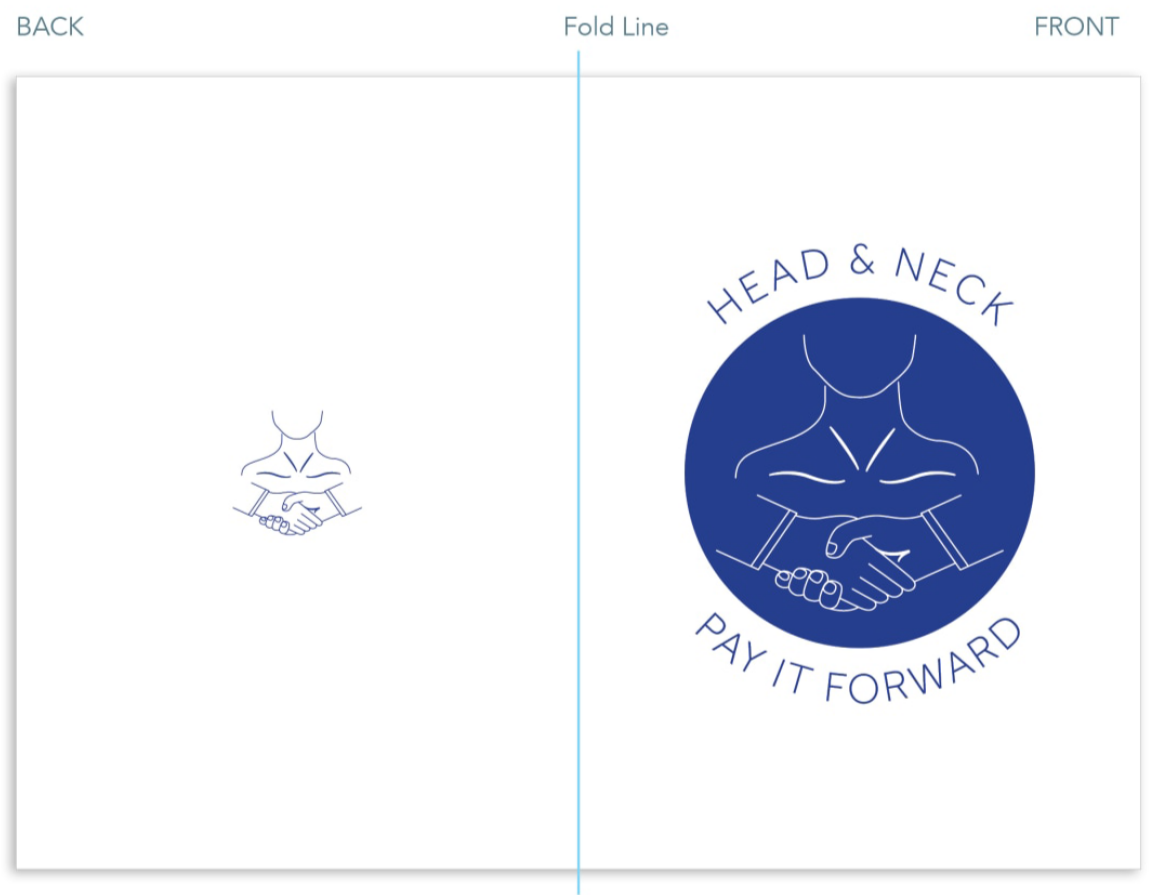


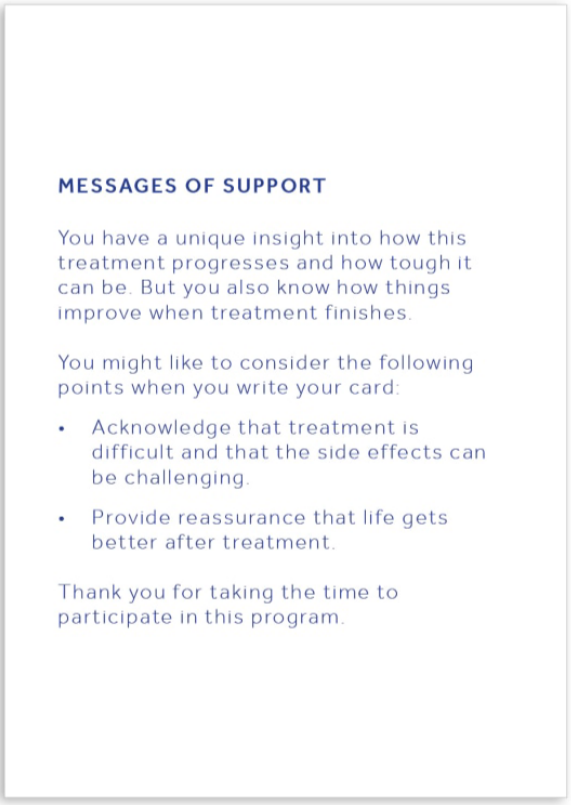


Supplementary file 2

**Pay It Forward Interview Guide**

You have been invited to partake in this interview because you participated in the Pay It Forward Head and Neck scheme, where you received a card from another patient, and you wrote a card for another patient. Today I’ll ask you a few questions about your experiences with this scheme and these cards. There are six main questions that I will ask you. I may also ask you a few further questions to seek more information or request clarification on the information you provide. This interview will be audio recorded to allow me to later transcribe and analyse what has been said. After I transcribe the interview, the audio recording will be deleted. Your answers will be anonymous, and you will only be identified by a participant ID number. Some people might find talking about their experiences with cancer distressing. If you feel this, please let me know. I can help with providing support as you need it, we can take a short break or stop the interview at any time. Do you have any questions before we start? I will start the recording now.

1. Tell me about your experience in receiving the card.
2. Tell me what you felt when you read the card?
   1. Prompt: did reading the card make you feel anything? Good or bad?
   2. Prompt: how did reading the card leave you feeling about the future?
   3. Prompt: what did reading the card make you feel about the patient who wrote the card?
   4. Prompt: did your feelings regarding the card change over time?
3. What did you do with the card?
   1. Prompt: did you read the card? If so, did you read it once or more than once?
   2. Prompt: did you show the card to anyone else?
   3. Prompt: did you display the card (OR keep it with you, OR store it, OR destroy it)?
4. What did you think when you were asked to write a card for another patient?
   1. Prompt: what kind of effect did writing the card have on how you felt?
   2. Prompt: what kind of impact did you think the card might have on another patient?
   3. Prompt: were you nervous about sharing your experience?
   4. Prompt: were you nervous about who might read it?
5. Tell me how you felt when you wrote a card?
   1. Prompt: did you find it easy or difficult to write the card?
   2. Prompt: how did you feel about sharing your experiences?
   3. Prompt: how did you feel about the card being anonymous?
   4. Prompt: after you had finished writing the card, did your feelings about it change either immediately or over the next few days or months?
6. Lastly, is there anything else you would like to tell us about receiving or writing the card?
   1. Prompt: you could tell us something else about your experience.
   2. Prompt: you could tell us something else about your participating in this scheme.
   3. Prompt: you could tell us if you have any thoughts on how the scheme could be improved for future patients.

Thank you. I will end the recording now.

Supplementary file 3

**Participant checking questions.**

Thank you for participating in the Pay it Forward study. The research team have conducted 13 interviews within this study. Interviews have been analysed, and two main themes have been identified. We would like to seek your feedback to ensure our interpretation is accurate.

- What is your study ID?

After reviewing and analysing the interviews about pay it forward the researchers identified two main themes:

1. Connectivity:
   - Participants described that when reading the card that it felt good to receive a message from someone who has been in the same situation.
   - A positive perception of the writer was often described.
   - When reading and/or writing the card participants often shared the process with family members.
   - Participants reported when creating their card, a need to ensure it did not cause harm to the next person
2. Benefit and value:
   - Some participants described that receiving the card gave them a sense of hope and encouragement that there was a 'light at the end of the tunnel'.
   - Participants often kept the card and re-read it at different points in the treatment and recovery journey.
   - When writing their card some participants reflected on the challenging time that they had overcome.

- How accurately do you feel the findings, overall, capture your thoughts and experiences?

Very Accurately

Somewhat accurately

Somewhat inaccurate

Very inaccurate

Unsure

- Is there anything you feel could be added to the findings to better reflect your personal experiences of the pay it forward initiative?
- Some participants spoke about the size of the card. Did you find the size of the pay it forward card appropriate or would you have preferred the card was larger or smaller?

Much smaller

Slightly smaller

Current size

Slightly larger

Much larger

- Pay it forward is currently in the format of a handwritten card. What would be your preference as to how you receive and pass on a message in this scheme?

Typed letter

Handwritten letter

Email

Handwritten Card

Audio message

Text message

Typed card

Other

- Some participants talked about the timing of receiving the card. Did you find the timing you received the pay it forward card appropriate or would you have preferred the card was received earlier or later?

The time that I received the card was good (week 5-6)

I would have preferred the card at the start of my treatment (week 1-2)

I would have preferred the card halfway through my treatment (week 3-4)

I would have preferred the card at the end of treatment (week 7)

- Did you find the timing when you wrote your pay it forward card appropriate or would you have preferred to write the card earlier or later?

The time that I wrote the card was good (week 5-6 after treatment)

I would have preferred to write the card straight after my treatment (week 1-2 after treatment)

I would have preferred the card to write the card later (3 to 6 months after my treatment)

- Pay it forward is currently anonymous. Do you prefer the process being anonymous?

Yes - I prefer to be anonymous.

No - I am comfortable being identifiable.

- The importance of carers and family members has been suggested in several interviews. The research team would like to seek the opinion of carers and family members about the pay it forward initiative through a short interview.

Would you be happy for us to contact you about this? Yes No

Thank you for your feedback and participation.
